# Relationship between serum iPTH and peritonitis episodes in patients undergoing continuous ambulatory peritoneal dialysis

**DOI:** 10.3389/fendo.2023.1081543

**Published:** 2023-03-27

**Authors:** Zihao Zhao, Qianqian Yan, Duopin Li, Guangpu Li, Jingjing Cai, Shaokang Pan, Jiayu Duan, Dongwei Liu, Zhangsuo Liu

**Affiliations:** ^1^ Department of Integrated Traditional and Western Nephrology, The First Affiliated Hospital of Zhengzhou University, Zhengzhou, China; ^2^ Research Institute of Nephrology, Zhengzhou University, Zhengzhou, China; ^3^ Henan Province Research Center for Kidney Disease, Zhengzhou, China; ^4^ Key Laboratory of Precision Diagnosis and Treatment for Chronic Kidney Disease in Henan Province, Zhengzhou, China

**Keywords:** continuous ambulatory peritoneal dialysis (CAPD), parathyroid hormone, peritonitis, end-stage renal disease (ESRD), hazard ratio (HR)

## Abstract

**Background:**

Peritonitis is considered as one of the most serious complications that cause hospitalization in patients undergoing continuous ambulatory peritoneal dialysis (CAPD). There is limited evidence on the impact of the parathyroid hormone (PTH) on the first peritoneal dialysis (PD)-associated peritonitis episode. We aimed to investigate the influence of serum intact parathyroid hormone (iPTH) on peritonitis in patients undergoing PD.

**Methods:**

This was a retrospective cohort study. Patients undergoing initial CAPD from a single center in China were enrolled. The baseline characteristics and clinical information were recorded. The primary outcome of interest was the occurrence of the first PD-associated peritonitis episode. Five Cox proportional hazard models were constructed in each group set. In group set 1, all participants were divided into three subgroups by tertiles of the serum concentration of iPTH; in group set 2, all participants were divided into three subgroups based on the serum concentration of iPTH with 150 pg/ml interval (<150, 150–300, and >300 pg/ml). Hazard ratios and 95% confidence intervals (CIs) were calculated for each model. The multivariate linear regression analysis elimination procedure assessed the association between the clinical characteristics at baseline and the iPTH levels. Restricted cubic spline models were constructed, and stratified analyses were also conducted.

**Results:**

A total of 582 patients undergoing initial PD (40% women; mean age, 45.1 ± 11.5 years) from a single center in China were recruited. The median follow-up duration was 25.3 months. Multivariate Cox regression analysis showed that, in the fully adjusted model, a higher serum iPTH level (tertile 3, iPTH >300 pg/ml) was significantly associated with a higher risk of PD-associated peritonitis at 3 years [tertile 3: hazard ratio (HR) = 1.53, 95%CI = 1.03–2.55, *p* = 0.03; iPTH > 300 pg/ml: HR = 1.57, 95%CI = 1.08–2.27, *p* = 0.02]. The hazard ratio for every 100 pg/ml increase in serum iPTH level was 1.12 (95%CI = 1.05–1.20, *p* < 0.01) in the total cohort when treating iPTH as a continuous variable.

**Conclusions:**

An elevated iPTH level was significantly associated with an increased risk of peritonitis in patients undergoing CAPD.

## Introduction

The high incidence of end-stage renal disease (ESRD) has become one of the major health problems worldwide (1, 2). Studies have estimated that 1.9 million people in Asia die prematurely every year due to lack of dialysis services (3, 4). Peritoneal dialysis is one of the renal replacement therapy methods for patients with ESRD (5). The semi-permeable membrane could transport uremic toxins from the bloodstream to the peritoneal dialysate, which is perfused into the abdominal cavity and refreshed routinely (6). One of the most important factors affecting the application of peritoneal dialysis (PD) is PD-associated peritonitis, which is a major infectious complication in patients undergoing PD and is usually accompanied by cloudy peritoneal effusion and abdominal pain (7, 8). Although a Peritoneal Dialysis Outcomes and Practice Patterns Study (PDOPPS) survey showed that the incidence of peritonitis varied among institutions within the same country (9), it was strongly associated with the risk of hospitalization, encapsulating peritoneal sclerosis, technical failure, and even death (10, 11). Poor patient compliance and a non-standard operation, which lead to bacteria entering through the air from the interface of the PD tube, are the suspected causal factors. However, previous studies have shown that a low immune status, such as hypoalbuminemia and vitamin D deficiency, and a self-inflammatory state are also closely related to PD-associated peritonitis (12–14).

The parathyroid hormone (PTH), a single-chain peptide hormone secreted by the main cells of the parathyroid gland, regulates the calcium levels in the body (15). It is composed of 84 amino acids, and the coding gene is located on the broken arm of chromosome 11. By stimulating the PTH receptor (PTHR), PTH controls the inorganic calcium matrix in the bone and plays a crucial role in the homeostasis of Ca^2+^ and phosphorus (16, 17). Serum PTH abnormalities are common in ESRD, particularly in patients undergoing dialysis. Previous studies have indicated that maintaining serum calcium, phosphorus, and intact PTH (iPTH) in healthy levels is beneficial for patients undergoing dialysis (18). However, from stage 3 chronic kidney disease (CKD) onwards, the renal excretion of phosphorus decreases; subsequently, the blood concentration of phosphorus and the PTH level increase from stage 3 of CKD (19, 20). Insufficient levels of 1,25(OH)2 vitamin D further decrease the absorption of intestinal calcium, elevate the PTH level, and result in secondary hyperparathyroidism (SHPT) (21, 22). The most obvious biochemical alteration of SHPT is the elevation of PTH, which becomes increasingly aggravated as CKD progresses (23). However, previous studies have rarely considered the iPTH level, an important factor in trial completion or termination.

To date, only a handful of studies have investigated the association between PTH and mortality in patients undergoing PD. Therefore, the purpose of the present study was to investigate the possible link between the baseline serum concentration of iPTH and new-onset peritonitis after adjusting for a variety of crucial variables and providing scientific evidence for iPTH treatment strategy in patients undergoing continuous ambulatory peritoneal dialysis (CAPD).

## Materials and methods

### Study population

This was a retrospective cohort study in a single center. Patients undergoing initial CAPD in the Department of Nephrology, The First Affiliated Hospital of Zhengzhou University, between January 2017 and October 2018 were enrolled in the present study. The exclusion criteria were as follows: 1) age less than 18 years; 2) comorbid malignancy; 3) comorbid severe underlying diseases such as primary respiratory or digestive system disease; and 4) peritonitis occurring within 3 months of dialysis initiation. All patients and caregivers received PD training at the hospital after catheter placement. The occurrence of peritonitis within 3 months was excluded to reduce bias due to improper handling at the commencement of dialysis. Accordingly, a total of 582 patients undergoing CAPD were finally recruited. This study was approved by the Research and Clinical Trial Ethics Committee of the First Affiliated Hospital of Zhengzhou University. All data were fully anonymized and all patient information collection procedures complied with the principle of confidentiality.

### Outcomes and measurements

The included patients were followed up for 3 years from the date of commencing CAPD. The primary outcome of interest was the occurrence of the first PD-associated peritonitis episode. At the end of follow-up, or death, transfer to hemodialysis and renal transplantation without previous peritonitis were censored. According to the International Society for Peritoneal Dialysis (ISPD) guidelines, PD peritonitis was diagnosed when at least two of the following were present: have clinical features such as abdominal pain and/or cloudy effluent; 2) dialysis effluent (intra-abdominal stay of at least 2 h) white blood cell (WBC) count >100 cells/μl, with >50% polymorphonuclear leukocytes; and 3) positive dialysate culture or Gram stain.

The baseline characteristics and clinical information of the whole cohort were retrieved from the Hospital Information System of The First Affiliated Hospital of Zhengzhou University. Patient characteristics were recorded at the initiation of PD, which included information on sex, age, systolic blood pressure (SBP), diastolic blood pressure (DBP), the primary cause of ESRD, body mass index (BMI), medication use, comorbidities, and smoking and drinking status. Medication use included erythropoietin (EPO), iron supplements, phosphorus-lowering drugs, uric acid-lowering drugs (e.g., allopurinol and febuxostat), PTH-regulating drugs (e.g., osteotriol, calcimimetics, and vitamin D analogs), and statins. The criteria for comorbidities were as follows: hypertension, diabetes mellitus (DM), cardiocerebral vascular disease (CVD) and edema confirmed by a physician with prescribing authority and documented medical history, and chronic hepatitis B confirmed by a documented medical history. The clinical parameters, which were measured at the laboratory of The First Affiliated Hospital of Zhengzhou University, included the following: WBC, neutrophil, lymphocyte, monocyte, eosinophil, basophil, and red blood cell (RBC) counts; hematocrit (Hct), hemoglobin (Hb), and platelet (PLT) counts; fasting blood glucose (FPG); CD4^+^, CD8^+^, and CD3^+^ counts; serum phosphorus (P), corrected calcium (cCa^2+^), potassium (K), sodium (Na), chloride (Cl), and magnesium (Mg) levels; carbon dioxide binding capacity (CO_2_); serum Fe level; unsaturated iron binding capacity (UIBC) and total iron binding capacity (TIBC); C-reactive protein (CRP); erythrocyte sedimentation rate (ESR); and the levels of uric acid (UA), total protein (TP), alanine aminotransferase (ALT), aspartate aminotransferase (AST), alkaline phosphatase (ALP), total bilirubin (Tbil), direct bilirubin (Dbil), total cholesterol (TC), triglycerides (TG), high-density lipoprotein (HDL), low-density lipoprotein (LDL), albumin (ALB), iPTH, blood urea nitrogen (BUN), and serum creatinine (Scr). Information on PD-related characteristics included laparoscopic insertion of PD catheter (yes or no), daily ultrafiltration, 24-h urine volume, total weekly *Kt*/*V*, and residual renal function (RRF). The parameters obtained in the echocardiography included right ventricular end dimension (RVD), interventricular septal thickness at diastole (IVSD), left ventricular end dimension (LVD), diameter of the left atrium (DLA), and ejection fraction (EF). Routine 12-lead electrocardiogram (ECG) included corrected QT (QTc), heart rate (HR), P–R interval (P.R), and ST- and T-wave (ST.T) changes.

The patients were divided into tertiles according to the serum iPTH levels in this cohort: tertile 1, ≤189 pg/ml; 189 pg/ml < tertile 2 < 340 pg/ml; and tertile 3, ≥340 pg/ml. Tertile 2 was used as a reference due to the mortality risk being lowest for this category. Moreover, the categories iPTH < 150 pg/ml, 150 pg/ml ≤ iPTH ≤ 300 pg/ml, and iPTH > 300 pg/ml, with iPTH 150–300 pg/ml as the reference, were also analyzed according to the recommendation of 2016 ISPD and the CORES study (24, 25). The baseline serum iPTH levels were treated as a categorical variable in the outcome analysis.

### Statistical analysis

All statistical analyses were performed using R software (version 4.1.2; R Project, www.r-project.org) and RStudio (version 1.4.1). Parameter values were presented as the mean ± standard deviation (SD) or median with interquartile range (IQR) for continuous variables, or as number (*n*) and percentage for categorical variables. A *p*-value <0.05 was considered as statistically significant.

The modeling time to the occurrence of the first episode of PD-associated peritonitis was performed using Kaplan–Meier survival analysis, and differences were analyzed using the log-rank test. The association between the baseline serum iPTH levels and the first PD-associated peritonitis episode was analyzed using Cox proportional hazards models, which were constructed using the baseline variables that were thought to be related to the outcomes chosen for five levels of confounding factor adjustments: model 1, adjusted for age, sex, BMI, and smoking and drinking status; model 2, adjusted for model 1 covariates plus comorbidities [hypertension, DM, CVD history, hepatitis B and edema, and medication use (e.g., EPO, iron supplements, phosphorus- and uric acid-lowering drugs, PTH-regulating drugs, and statins)]; model 3, adjusted for model 2 covariates plus the dialysis-related parameters including ultrafiltration volume, 24-h urine output, laparoscopy, *Kt*/*V*, and RRF; model 4, adjusted for model 3 covariates plus the laboratory variables including WBC, RBC, Hb, PLT, GLU, CD4, CD8, CD3, ESR, CPR, Na, Cl, Ca, P, Mg, CO_2_, BUN, K, Fe, UIBC, TIBC, Scr, UA, ALB, ALT, AST, ALP, TP, Tbil, Dbil, TC, TG, HDL, and LDL; and model 5, adjusted for model 4 covariates plus RVD, IVSD, LVD, DLA, EF, QTc, HR, P.R, and ST.T changes. The results were presented as hazard ratios (HRs) and 95% confidence intervals (CIs). Multivariate linear regression analysis with a stepwise elimination procedure assessed the associations between the clinical characteristics at baseline and iPTH. To strengthen the findings in order to examine the relationship between iPTH level and peritonitis, analyses of the continuous variables were conducted, with HRs expressed per 100-pg/ml and per 1-SD higher iPTH level. Two sensitivity analyses were also conducted. All of the patients in the cohort were dichotomized into men or women and with or without PTH-regulating drugs for repeated Cox regression analysis. Furthermore, we examined iPTH as a continuous predictor using restricted cubic spline models (26) based on the fully adjusted Cox proportional hazards model. In addition, we also conducted stratified analyses based on potentially relevant markers, including BUN, Scr, ALB, phosphorus, cCa^2+^, and TIBC, and plotted forest plots using the “forestplot” package in R.

## Results

### Patient characteristics

**Figure 1:**
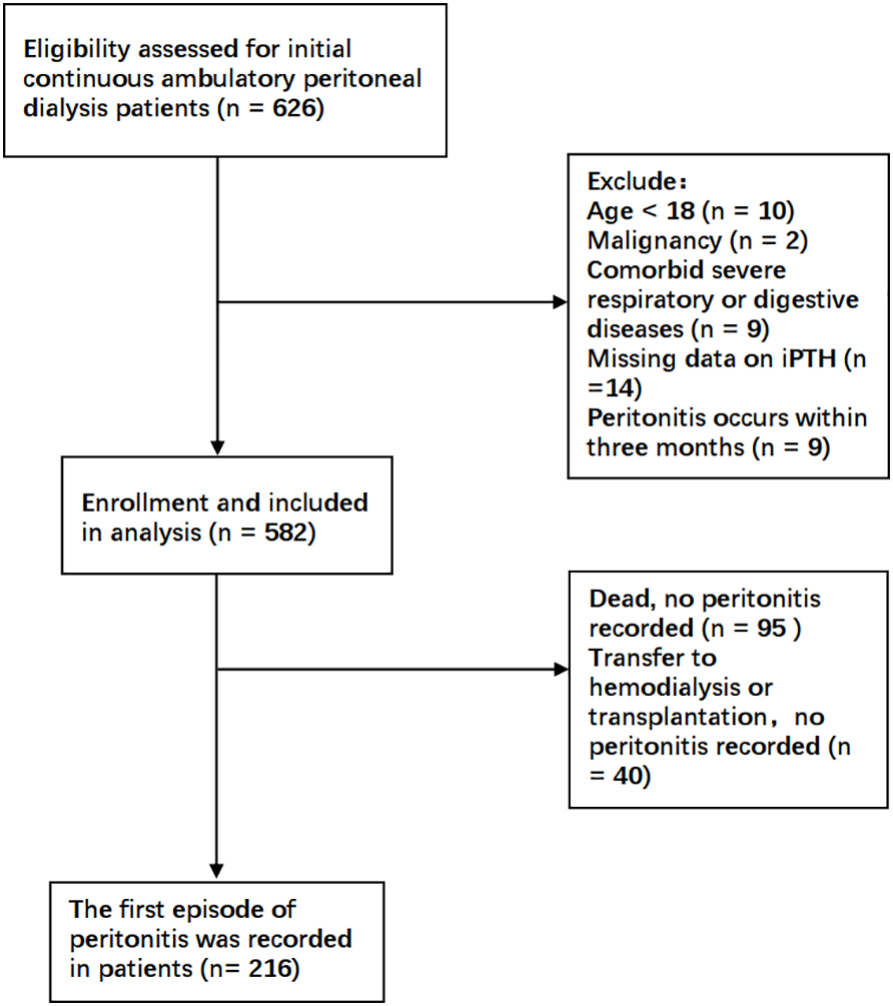
Flowchart for patient selection.

As shown in **Figure 1**, a total of 582 patients undergoing CAPD were enrolled in the present study. **Table 1** and **Supplementary Table S1** detail the baseline demographic and clinical characteristics of all patients and each subgroup, along with the whole cohort. The mean age of the patients was 45.1 ± 11.5 years, with 40% women (*n* = 233), 12.2% with diabetes (*n* = 71), and with a median follow-up period of 25.3 months. For all patients, the mean serum iPTH level was 310.0pg/ml (range, 8.2–2760 pg/ml). A total of 346 participants (59.5%) were taking PTH-regulating drugs at baseline. Compared to patients with iPTH levels in the lowest tertile, those with higher serum iPTH levels were more likely to have higher BMI and be men, as well as have a higher prevalence of hypertension and edema. The baseline serum creatinine and phosphorus levels increased, while the cCa^2+^ level and RBC and Hb counts decreased in the higher tertiles. The other characteristics were not significantly different between the three tertiles. There were 126 patients who had PD-associated peritonitis during the follow-up period, including 90 that occurred within 1 year of initiating PD.

**Table 1:** Baseline demographic and clinical characteristic of the study population by serum intact parathyroid hormone (iPTH) tertile.

	Total (*N* = 582)	Tertile 1 (*N* = 194)	Tertile 2 (*N* = 194)	Tertile 3 (*N* = 194)	*p*-value
Sex, male	349 (60.0%)	109 (56.2%)	123 (63.4%)	117 (60.3%)	0.35
Age (years)	45.1 ± 11.5	46.4 ± 11.4	46.7 ± 11.1	42.1 ± 11.4	<0.01
SBP (mmHg)	140 ± 18.2	137 ± 18.6	140 ± 15.4	143 ± 19.9	0.01
DBP (mmHg)	87 ± 12.4	85.5 ± 12.2	86.8 ± 11.0	88.7 ± 13.7	0.04
Smoker, yes	47 (8.1%)	19 (9.8%)	14 (7.2%)	14 (7.2%)	0.56
Drinker, yes	32 (5.5%)	9 (4.6%)	13 (6.7%)	10 (5.2%)	0.65
BMI (kg/m^2^)	23.4 ± 4.83	22.8 ± 4.81	23.2 ± 4.70	24.3 ± 4.87	<0.01
Comorbidities					
Hypertension, *n*(%)	507 (87.1%)	162 (83.5%)	179 (92.3%)	166 (85.6%)	0.03
Diabetes, *n*(%)	71 (12.2%)	29 (14.9%)	14 (7.2%)	28 (14.4%)	0.03
CVD, *n*(%)	97 (16.7%)	38 (19.6%)	27 (13.9%)	32 (16.5%)	0.32
Hepatitis B, *n*(%)	35 (6.0%)	11 (5.7%)	10 (5.2%)	14 (7.2%)	0.67
Edema, *n*(%)	278 (47.8%)	81 (41.8%)	91 (46.9%)	106 (54.6%)	0.04
Medication use					
EPO (%)	436 (74.9%)	142 (73.2%)	149 (76.8%)	145 (74.7%)	0.71
Iron supplements (%)	428 (73.5%)	138 (71.1%)	144 (74.2%)	146 (75.3%)	0.63
Phosphorus-lowering drugs (%)	374 (64.3%)	145 (74.7%)	115 (59.3%)	114 (58.8%)	<0.01
PTH-regulating drugs (%)	346 (59.5%)	95 (49.0%)	101 (52.1%)	150 (77.3%)	<0.01
UA-lowering drugs (%)	102 (17.5%)	42 (21.6%)	31 (16.0%)	29 (14.9%)	0.17
Statins (%)	56 (9.6%)	14 (7.2%)	25 (12.9%)	17 (8.8%)	0.15
Dialysis-related data					
Laparoscopy (%)	372 (63.9%)	113 (58.2%)	126 (64.9%)	133 (68.6%)	0.10
Ultrafiltration (ml/day)	400 (−1,500 to 2,500)	420 (−900 to 2,500)	400 (−800 to 2,000)	400 (−1,500 to 2,300)	0.86
24-h urine output (L)	200 (0–2,100)	200 (0–2,100)	200 (0–2,100)	200 (0–2,100)	0.71
Total weekly *Kt*/*V*	2.09 ± 0.31	2.11 ± 0.32	2.07 ± 0.29	2.09 ± 0.31	0.47
RRF (ml/min/1.73 m^2^)	3.59 ± 1.57	3.5 ± 1.48	3.65 ± 1.68	3.62 ± 1.55	0.60
Laboratory variables					
WBC count (×10^9^/L)	6.14 ± 1.99	6.32 ± 2.32	6.12 ± 1.75	5.98 ± 1.84	0.25
RBC count (×10^12^/L)	3.33 ± 0.80	3.52 ± 0.82	3.25 ± 0.7	3.37 ± 0.78	<0.01
Hemoglobin (g/L)	102 ± 22.6	106 ± 22.7	101 ± 23.3	98.5 ± 21.1	<0.01
Platelet (×10^9^/L)	190 ± 69.3	195 ± 70.2	186 ± 71.3	189 ± 66.2	0.41
FPG (mmol/L)	4.94 ± 2.2	4.91 ± 2.07	4.93 ± 2.46	4.97 ± 2.04	0.96
Phosphorus (mmol/L)	1.76 ± 0.51	1.63 ± 0.49	1.82 ± 0.53	1.84 ± 0.47	<0.01
Corrected calcium (mmol/L)	2.15 ± 0.26	2.25 ± 0.28	2.1 ± 0.25	2.09 ± 0.23	<0.01
Potassium (mmol/L)	4.31 ± 0.74	4.30 ± 0.74	4.36 ± 0.79	4.28 ± 0.70	0.53
Serum Fe (μmol/L)	14.5 ± 6.68	14.2 ± 7.04	14.3 ± 5.9	14.9 ± 7.06	0.60
CRP (mg/L)	13.4 ± 31.4	14.5 ± 32.8	13 ± 31	12.7 ± 30.4	0.82
iPTH (pg/ml)	262 (8.20–2,760)	110 (8.20–189)	262 (189–339)	478 (340–2,760)	<0.01
BUN (mmol/L)	23.2 ± 8.6	21.9 ± 7.89	24.1 ± 9.63	23.7 ± 8.06	0.03
Creatinine (mg/dl)	10.65 ± 4.32	836 ± 289	979 ± 478	999 ± 329	<0.01
Uric acid (mmol/L)	375 ± 95.9	368 ± 108	385 ± 94.8	372 ± 83.5	0.21
Albumin (g/L)	35.9 ± 6.25	34.8 ± 6.46	35.9 ± 6.16	36.9 ± 5.97	0.01
Cholesterol (mmol/L)	4.46 ± 1.74	4.56 ± 1.92	4.28 ± 1.04	4.54 ± 2.06	0.22
Triglycerides (mmol/L)	1.43 ± 0.84	1.44 ± 0.88	1.4 ± 0.79	1.44 ± 0.84	0.84
HDL (mmol/L)	1.13 ± 0.34	1.12 ± 0.32	1.11 ± 0.35	1.15 ± 0.36	0.52
LDL (mmol/L)	2.81 ± 0.92	2.85 ± 0.98	2.74 ± 0.86	2.84 ± 0.91	0.45
ECG and echocardiography					
EF (%)	61.1 ± 5.36	61.7 ± 4.57	61.4 ± 5.45	60.3 ± 5.92	0.03
QT interval (ms)	392 ± 57.8	393 ± 40.6	386 ± 72.9	396 ± 55.1	0.23
HR (bpm)	77.3 ± 12.4	76.4 ± 11.7	78.2 ± 13.1	77.5 ± 12.5	0.37
ST.T changes, yes (%)	210 (36.1%)	72 (37.1%)	63 (32.5%)	75 (38.7%)	0.42

SBP, systolic blood pressure; DBP, diastolic blood pressure; BMI, body mass index; CVD, cardiocerebral vascular disease history; EPO, erythropoietin; UA, uric acid; RRF, residual renal function; WBC, white blood cell; RBC, red blood cell; FPG, fasting blood glucose; CRP, C-reactive protein; iPTH, intact parathyroid hormone; BUN, blood urea nitrogen; HDL, high-density lipoprotein; LDL, low-density lipoprotein; EF, ejection fraction; HR, heart rate.

### iPTH and the occurrence of the first PD-associated peritonitis episode

**Figure 2:**
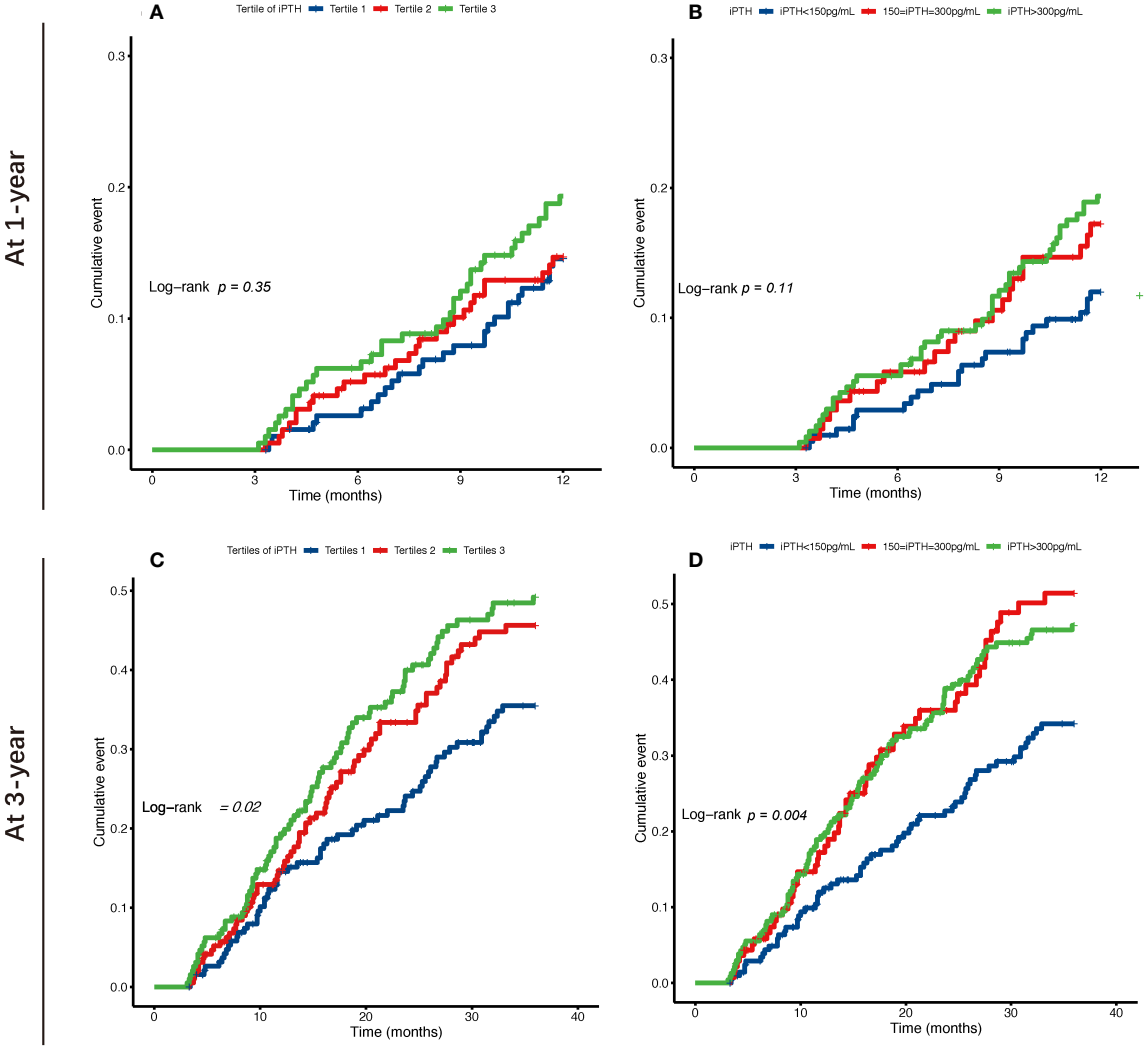
Kaplan–Meier survival curves of the risk of occurrence of first peritonitis episode at 1 and 3 years for tertiles **(A, C)** and categories by 150–300 pg/ml **(B, D)** of the serum intact parathyroid hormone (iPTH) levels in patients undergoing continuous ambulatory peritoneal dialysis.

The Kaplan–Meier cumulative incidence curves are shown in **Figure 2**, and the survival curves with a risk table are plotted in **Supplementary Figure S1**. The lower and higher iPTH level groups showed a significantly increased risk of occurrence of first peritonitis episode at 3 years in both classification categories (log-rank test: *p* = 0.02 and *p* = 0.004, respectively), while the highest iPTH level group showed a significantly increased 1-year cumulative risk of occurrence of first peritonitis episode.

The associations of the tertiles and different iPTH level groups with the risk of first peritonitis episode by Cox regression analysis are shown in **Tables 2**, **3**. Compared with iPTH tertile 2, iPTH tertile 3 was associated with a higher cumulative risk of peritonitis episode in the fully adjusted Cox model [model 5: adjusted HR (aHR) = 1.90, 95%CI = 1.02–3.53, *p* = 0.04, at 1 year; aHR = 1.48, 95%CI = 1.01–2.17, *p* = 0.05, at 3 years] (**Table 2**). In the multivariate fully adjusted model, the iPTH > 300 pg/ml group *versus* the 150 pg/ml < iPTH < 300 pg/ml group showed significant association with the risk of first peritonitis episode at 1 year (aHR = 2.35, 95%CI = 1.30–4.26, *p *< 0.01) and at 3 years (aHR = 1.56, 95%CI = 1.08–2.26, *p* = 0.02) (**Table 3**).

**Table 2:** Cox regression models of the first peritonitis episode for serum intact parathyroid hormone (iPTH) level (tertiles as categories).

	iPTH tertiles	First peritonitis episode at 1 year		First peritonitis episode at 3 years	
		HR (95% CI)	*p*-value	HR (95% CI)	*p*-value
Unadjusted	Tertile 2	1 (as reference)		1 (as reference)	
	Tertile 1	1.04 (0.61–1.77)	0.89	1.39 (0.99–1.96)	0.06
	Tertile 3	1.39 (0.84–2.29)	0.20	1.59 (1.14–2.21)	0.01
Model 1	Tertile 2	1 (as reference)		1 (as reference)	
	Tertile 1	1.03 (0.60–1.76)	0.92	1.40 (0.99–1.98)	0.05
	Tertile 3	1.29 (0.77–2.15)	0.33	1.56 (1.11–2.19)	0.01
Model 2	Tertile 2	1 (as reference)		1 (as reference)	
	Tertile 1	1.06 (0.61–1.82)	0.85	1.41 (0.99–2.00)	0.06
	Tertile 3	1.27 (0.75–2.14)	0.38	1.40 (0.99–2.00)	0.05
Model 3	Tertile 2	1 (as reference)		1 (as reference)	
	Tertile 1	1.04 (0.60–1.80)	0.89	1.39 (0.98–1.99)	0.07
	Tertile 3	1.30 (0.77–2.20)	0.33	1.44 (1.02–2.05)	0.04
Model 4	Tertile 2	1 (as reference)		1 (as reference)	
	Tertile 1	0.81 (0.44–1.48)	0.49	1.25 (0.85–1.85)	0.26
	Tertile 3	1.96 (1.07–3.56)	0.03	1.44 (0.98–2.10)	0.06
Model 5	Tertile 2	1 (as reference)		1 (as reference)	
	Tertile 1	0.80 (0.43–1.48)	0.48	1.30 (0.87–1.94)	0.21
	Tertile 3	1.94 (1.04–3.61)	0.04	1.53 (1.03–2.25)	0.03

Tertile 2 of iPTH was taken as the reference group. Model 1: adjusted for age, sex, BMI, and smoking and drinking status. Model 2: adjusted for model 1 covariates plus comorbidities (hypertension, DM, CVD history, hepatitis B and edema, and medication use (e.g., EPO, iron supplements, phosphorus- and uric acid-lowering drugs, PTH-regulating drugs, and statins). Model 3: adjusted for model 2 covariates plus ultrafiltration volume, 24-h urine output, laparoscopy, Kt/V, and RRF. Model 4: adjusted for model 3 covariates plus WBC, RBC, Hb, PLT, GLU, CD4, CD8, CD3, ESR, CPR, Na, Cl, cCa^2+^, P, Mg, CO_2_, BUN, K, Fe, UIBC, TIBC, Scr, UA, ALB, ALT, AST, ALP, TP, Tbil, Dbil, TC, TG, HDL, and LDL. Model 5: adjusted for model 4 covariates plus RVD, IVSD, LVD, DLA, EF, QTc, HR, P.R, and ST.T changes.

HR, hazard ratio; CI, confidence interval.

**Table 3:** Cox regression models of the first peritonitis episode for serum intact parathyroid hormone (iPTH) level (150 and 300 pg/ml as categories).

	iPTH (pg/ml)	First peritonitis episode at 1 year		First peritonitis episode at 3 years	
		HR (95% CI)	*p*-value	HR (95% CI)	*p*-value
Unadjusted	150 ≤ iPTH ≤ 300	1 (as reference)		1 (as reference)	
	iPTH < 150	1.50 (0.84–2.67)	0.17	1.71 (1.19–2.46)	0.00
	iPTH > 300	1.70 (1.03–2.79)	0.04	1.60 (1.16–2.20)	0.00
Model 1	150 ≤ iPTH ≤ 300	1 (as reference)		1 (as reference)	
	iPTH < 150	1.49 (0.83–2.66)	0.18	1.71 (1.19–2.47)	0.00
	iPTH > 300	1.58 (0.96–2.63)	0.07	1.54 (1.12–2.13)	0.01
Model 2	150 ≤ iPTH ≤ 300	1 (as reference)		1 (as reference)	
	iPTH < 150	1.52 (0.84–2.74)	0.17	1.65 (1.13–2.41)	0.01
	iPTH > 300	1.55 (0.92–2.62)	0.10	1.39 (0.99–1.95)	0.01
Model 3	150 ≤ iPTH ≤ 300	1 (as reference)		1 (as reference)	
	iPTH < 150	1.48 (0.81–2.70)	0.20	1.59 (1.09–2.33)	0.02
	iPTH > 300	1.59 (0.94–2.68)	0.09	1.41 (1.01–1.97)	0.05
Model 4	150 ≤ iPTH ≤ 300	1 (as reference)		1 (as reference)	
	iPTH < 150	1.33 (0.68–2.61)	0.40	1.56 (1.03–2.38)	0.04
	iPTH > 300	2.51 (1.40–4.52)	0.00	1.54 (1.07–2.23)	0.02
Model 5	150 ≤ iPTH ≤ 300	1 (as reference)		1 (as reference)	
	iPTH < 150	1.26 (0.64–2.48)	0.50	1.57 (1.02–2.40)	0.04
	iPTH > 300	2.34 (1.29–4.24)	0.01	1.57 (1.08–2.27)	0.02

Tertile 2 of iPTH was taken as the as reference group. Model 1: adjusted for age, sex, BMI, and smoking and drinking status. Model 2: adjusted for model 1 covariates plus comorbidities (hypertension, DM, CVD history, hepatitis B and edema, and medication use (e.g., EPO, iron supplements, phosphorus- and uric acid-lowering drugs, PTH-regulating drugs, and statins). Model 3: adjusted for model 2 covariates plus ultrafiltration volume, 24-h urine output, laparoscopy, Kt/V, and RRF. Model 4: adjusted for model 3 covariates plus WBC, RBC, Hb, PLT, GLU, CD4, CD8, CD3, ESR, CPR, Na, Cl, cCa^2+^, P, Mg, CO_2_, BUN, K, Fe, UIBC, TIBC, Scr, UA, ALB, ALT, AST, ALP, TP, Tbil, Dbil, TC, TG, HDL, and LDL. Model 5: adjusted for model 4 covariates plus RVD, IVSD, LVD, DLA, EF, QTc, HR, P.R, and ST.T changes.

HR, hazard ratio; CI, confidence interval.

We used Pearson’s correlation analysis to confirm that there was no significant correlation (|*r*| > 0.6) between every two baseline clinical data variables, except for the combination of hypertension and diabetes (**Supplementary Figure S2**). Moreover, as shown in **Table 4**, the baseline iPTH level was positively correlated with PTH-regulating drug use, edema, and the ALB, ALP, Na, P, and TIBC levels and negatively correlated with age, smoking status, Cl, Ca, Mg, UA, AST, and Dbil levels, and ST.T changes in the multivariate linear regression analysis.

**Table 4:** Multivariate linear regression of the intact parathyroid hormone (iPTH) levels with clinical parameters.

	Estimate	SE	*t*	*p*-value
Age	−2.087	0.827	−2.524	0.012
Smoker (yes *vs*. no)	−71.970	34.306	−2.098	0.036
Use of PTH drug (yes *vs*. no)	67.072	19.325	3.471	0.001
Combined edema (yes *vs*. no)	39.220	18.793	2.087	0.037
Albumin	5.993	1.936	3.096	0.002
Alkaline phosphate	0.755	0.140	5.390	0.000
Serum Na	7.647	2.801	2.730	0.007
Serum Cl	−7.013	2.262	−3.101	0.002
cCa^2+^	−277.438	40.655	−6.824	0.000
Serum phosphorus	58.024	20.245	2.866	0.004
Serum Mg	−162.446	59.497	−2.730	0.007
Uric acid	−0.211	0.104	−2.036	0.042
AST	−2.188	0.895	−2.444	0.015
Dbil	−13.304	4.772	−2.788	0.005
TIBC	3.282	1.296	2.532	0.012
ST.T changes (yes or no)	−47.030	19.487	−2.413	0.016

SE, standard error; cCa^2+^, corrected calcium; AST, aspartate aminotransferase; Dbil, direct bilirubin; TIBC, total iron binding capacity.

### Sensitivity and stratified analysis

The association of the serum iPTH concentration with the first peritonitis episode, treating iPTH as a continuous variable, was examined using multivariate fully adjusted Cox models. As shown in **Table 5**, in the total cohort, the aHRs for every 100-pg/ml increase in iPTH level were 1.12 (95%CI = 1.05–1.20, *p* < 0.01, at 3 years) and 1.19 (95%CI = 1.10–1.29, *p* < 0.01, at 1 year), while those for every 1-SD increase were 1.34 (95%CI = 1.14–1.58, *p* < 0.01, at 3 years) and 1.56 (95%CI = 1.28–1.88, *p* < 0.01, at 1 year). With each 100-pg/ml and 1-SD increase in iPTH level, similar associations were found in men (aHR = 1.25, 95%CI = 1.13–1.39, *p* < 0.01, at 1 year; aHR = 1.22, 95%CI = 1.13–1.31, *p* < 0.01, at 3 years) and in patients taking PTH-regulating drugs (aHR = 1.29, 95%CI = 1.18–1.42, *p* < 0.01, at 1 year; aHR = 1.22, 95%CI = 1.13–1.31, *p* < 0.01, at 3 years).

**Table 5:** First peritonitis episode for each 100-pg/ml and 1-SD increase in the serum intact parathyroid hormone (iPTH) level.

	Outcome	For each 100-pg/ml increase in iPTH level		For each 1-SD increase in iPTH level	
		HR (95% CI)	*p*-value	HR (95% CI)	*p*-value
Total cohort (*n* = 582)	1 year	1.19 (1.10–1.29)	<0.01	1.56 (1.28–1.88)	<0.01
	3 years	1.12 (1.05–1.20)	<0.01	1.34 (1.14–1.58)	<0.01
Men (*n* = 349)	1 year	1.25 (1.13–1.39)	<0.01	1.76 (1.36–2.27)	<0.01
	3 years	1.22 (1.13–1.31)	<0.01	1.64 (1.36–1.98)	<0.01
Women (*n* = 233)	1 year	1.13 (0.90–1.43)	0.29	1.37 (0.76–2.44)	0.29
	3 years	1.07 (0.94–1.21)	0.30	1.18 (0.87–1.61)	0.30
Excluding taking PTH drug (*n* = 236)	1 year	1.40 (0.86–2.28)	0.17	2.33 (0.69–7.81)	0.17
	3 years	1.07 (0.89–1.28)	0.47	1.13 (0.72–1.78)	0.60
Patients taking PTH drug (*n* = 346)	1 year	1.29 (1.18–1.42)	<0.01	1.88 (1.50–2.37)	<0.01
	3 years	1.22 (1.13–1.31)	<0.01	1.64 (1.36–1.98)	<0.01

Adjusted for the model 5 covariates in **Table 2**.

SD, standard deviation; HR, hazard ratio; 95%CI, 95% confidence interval.

**Figure 3:**
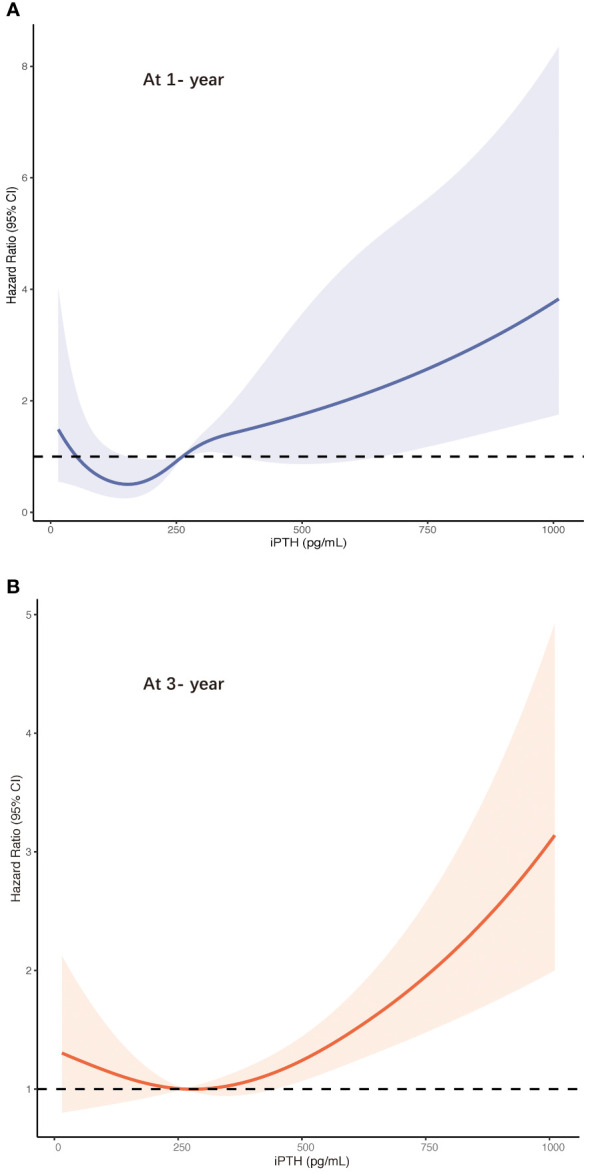
Risk of first peritonitis episode according to serum intact parathyroid hormone ((iPTH) levels with the fully adjusted (model 5) restricted cubic spline models at 1 year **(A)** and at 3 years **(B)**. *CI*, confidence interval.

Furthermore, these associations were validated in the restricted cubic spline model analyses. The results are summarized in **Figure 3**. In contrast to the peritonitis risk for lower iPTH levels not reaching statistical significance, an increase in the peritonitis risk was more distinguished in the higher iPTH levels with a narrow CI range. The results leaned toward a more J-shaped association between the levels of iPTH and the risk of first PD-related peritonitis episode after adjusting for multiple confounding factors in patients undergoing PD. The CI values in the cubic spline curves may indicate that an increase in the risk of peritonitis episodes at 1 and 3 years was more pronounced in the higher than about 600 and 400 pg/ml iPTH levels, respectively.

**Figure 4:**
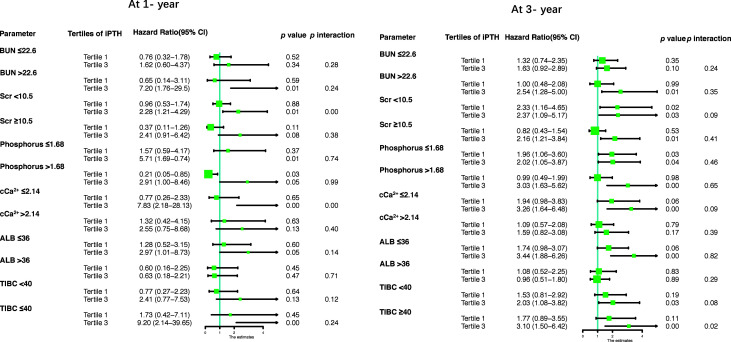
Forest plots for stratified analyses. *BUN*, blood urea nitrogen; *Scr*, serum creatinine; *cCa^2+^*, corrected calcium; *ALB*, albumin; *TIBC*, total iron binding capacity.

The results of the stratified analyses based on the BUN, Scr, phosphorus, cCa^2+^, ALB, and TIBC levels are shown in **Table 6** and **Figure 4**. The cohort was subdivided into two subgroups based on the median of these parameters. We found that Scr (<10.5 mg/dl) and cCa^2+^ (≤2.14 mmol/L) modified the association between iPTH and the risk of first peritonitis episode at 1 year (both *p* < 0.01 for interaction), while TIBC (≤40 μmol/L) modified the risk association at 3 years (*p* < 0.02 for interaction). The other *p*-values for subgroup interaction revealed non-significant results, implying that there were no significant interaction effects between iPTH and these clinical markers.

**Table 6:** Stratified analyses based on various clinical markers.

Parameter	iPTH tertiles	1-year cumulative peritonitis risk			3-year cumulative peritonitis risk		
		HR (95% CI)	*p*-value	*p* interaction	HR (95% CI)	*p-*value	*p* interaction
BUN ≤ 22.6	Tertile 1	0.76 (0.32–1.78)	0.52		1.32 (0.74–2.35)	*0.35*	
	Tertile 3	1.62 (0.60–4.37)	0.34	0.28	1.63 (0.92–2.89)	*0.10*	0.24
BUN > 22.6	Tertile 1	0.65 (0.14–3.11)	0.59		1.00 (0.48–2.08)	*0.99*	
	Tertile 3	7.20 (1.76–29.5)	0.01	0.24	2.54 (1.28–5.00)	*0.01*	0.35
Scr < 10.5	Tertile 1	0.96 (0.53–1.74)	0.88		2.33 (1.16–4.65)	*0.02*	
	Tertile 3	2.28 (1.21–4.29)	0.01	0.00	2.37 (1.09–5.17)	*0.03*	0.09
Scr ≥ 10.5	Tertile 1	0.37 (0.11–1.26)	0.11		0.82 (0.43–1.54)	*0.53*	
	Tertile 3	2.41 (0.91–6.42)	0.08	0.38	2.16 (1.21–3.84)	*0.01*	0.41
Phosphorus ≤ 1.68	Tertile 1	1.57 (0.59–4.17)	0.37		1.96 (1.06–3.60)	*0.03*	
	Tertile 3	5.71 (1.69–0.74)	0.01	0.74	2.02 (1.05–3.87)	*0.04*	0.46
Phosphorus > 1.68	Tertile 1	0.21 (0.05–0.85)	0.03		0.99 (0.49–1.99)	*0.98*	
	Tertile 3	2.91 (1.00–8.46)	0.05	0.99	3.03 (1.63–5.62)	*0.00*	0.65
cCa^2+^ ≤ 2.14	Tertile 1	0.77 (0.26–2.33)	0.65		1.94 (0.98–3.83)	*0.06*	
	Tertile 3	7.83 (2.18–28.13)	0.00	0.00	3.26 (1.64–6.48)	*0.00*	0.09
cCa^2+^ > 2.14	Tertile 1	1.32 (0.42–4.15)	0.63		1.09 (0.57–2.08)	*0.79*	
	Tertile 3	2.55 (0.75–8.68)	0.13	0.40	1.59 (0.82–3.08)	*0.17*	0.39
ALB ≤ 36	Tertile 1	1.28 (0.52–3.15)	0.60		1.74 (0.98–3.07)	*0.06*	
	Tertile 3	2.97 (1.01–8.73)	0.05	0.14	3.44 (1.88–6.26)	*0.00*	0.82
ALB > 36	Tertile 1	0.60 (0.16–2.25)	0.45		1.08 (0.52–2.25)	*0.83*	
	Tertile 3	0.63 (0.18–2.21)	0.47	0.71	0.96 (0.51–1.80)	*0.89*	0.29
TIBC < 40	Tertile 1	0.77 (0.27–2.23)	0.64		1.53 (0.81–2.92)	*0.19*	
	Tertile 3	2.41 (0.77–7.53)	0.13	0.12	2.03 (1.08–3.82)	*0.03*	0.08
TIBC ≥ 40	Tertile 1	1.73 (0.42–7.11)	0.45		1.77 (0.89–3.55)	*0.11*	
	Tertile 3	9.20 (2.14–39.65)	0.00	0.24	3.10 (1.50–6.42)	*0.00*	0.02

Tertile 2 of iPTH was taken as the reference group.

HR, hazard ratio; 95% CI, 95% confidence interval; p interaction: p value for the interaction analysis; BUN, blood urea nitrogen; Scr, serum creatinine; cCa^2+^, corrected calcium; ALB, albumin; TIBC, total iron binding capacity.

## Discussion

In this retrospectively cohort study, we found that elevated serum iPTH levels were associated with a higher risk of the occurrence of first PD-associated peritonitis episode in patients undergoing initial CAPD, independent of multiple confounding factors such as demographic characteristics, comorbidities, and laboratory variables. Every 100-pg/ml elevation of the iPTH level at baseline was also significantly associated with a higher risk of the development of peritonitis at 1 and 3 years. The restricted cubic spline models further confirmed these results. A significant association remained particularly in men or after excluding the patients taking PTH-regulating agents.

The occurrence and consequences of PD-associated peritonitis, which is associated with a higher mortality risk and is the leading cause of technique failure among patients receiving PD, increased the cost of treatment and restricted the widespread utilization of PD (10). However, peritonitis is still a common and serious complication in patients undergoing PD (27, 28). A study involving multiple countries showed that the crude rate of peritonitis was 0.28 episode/patient-year; however, there is a low overall peritonitis cure rate, ranging between 54% and 68%, among all participating countries (29). Peritonitis frequently results in a decreased peritoneal ultrafiltration capacity and is the most common cause of transfer to long-term hemodialysis (30). Previous studies have identified common risk factors for peritonitis, such as exposure to dialysis fluid and catheters and touch contamination, and the preventive measures are mainly the use of prophylactic antibiotics before PD catheter insertion, daily topical application of antibiotic cream to the catheter exit site, and training and nursing practice (31–35). Risk factors including demographic characteristics such as gender and age, comorbidities such as diabetes, and laboratory indicators such as albumin have also been pointed out and summarized in previous studies (36–39).

PTH consists of 84 amino acids that are secreted after it is cleaved from the preproparathyroid hormone (115 amino acids) to the proparathyroid hormone (90 amino acids). The active biological form is the intact PTH (1–84), which plays a major role in regulating calcium metabolism (40). In a physiological state, PTH generates a high bone turnover state and the release of calcium from the skeleton, upregulating the *CYP27B1* (1α-hydroxylase) gene and inhibiting phosphate reabsorption in the proximal tubules of the kidney, as well as converting the circulating form of vitamin D, 25-hydroxy vitamin D [25(OH)D], into the active form, 1,25(OH)2D3 (40, 41). In CKD, and especially in patients on dialysis, SHPT manifested by an elevated serum PTH is a particularly common complication (23). With the reduction in kidney function, there is a preferential increase in serum PTH, leading to the increased expression of fibroblast growth factor 23 (FGF-23). On the one hand, FGF-23, which has been shown to have intact activity in patients undergoing PD, can increase the expression and secretion of inflammatory factors (42, 43) and has been reported to activate local inflammation in organs *via* nuclear factor of activated T cells, FGF receptor 4 (FGFR4)/phospholipase C gamma (PLCγ), and other pathways (44), all of which increase its potential association with peritonitis. On the other hand, increased skeletal resistance to PTH results in osteodystrophy in CKD, and continued development of SHPT leads to hyperphosphatemia, vascular and organ calcification, and an increased risk of all-cause mortality (45–47). Interestingly, the latest research found that the all-cause mortality with low PTH is similar to that of patients with SHPT undergoing hemodialysis (17), that a low serum iPTH level is an independent predictor of infection-related mortality in incident dialysis patients (48), and that even combinations of low iPTH with other specific indicators are independently associated with increased all-cause and cardiovascular mortality in patients undergoing PD (49, 50). For dialysis patients, the phase changes in the levels of serum PTH, calcium, and phosphorus are more complex and are closely related to the emergence of CKD–mineral and bone disorder and adjustments in the treatment regimens (47, 51). Treatment with oral PTH-lowering vitamin D or its analogs has been shown to protect against peritoneal remodeling on PD and even reduce the risk of peritonitis (12, 52). Sevelamer, which belongs to another class of drugs, may be beneficial in reducing the endotoxin levels in dialysis patients and in improving the endothelial function and inflammatory response in patients undergoing PD, despite studies showing that it is not associated with a higher risk of peritonitis (53, 54). However, there are still no definitive accepted explanations on whether increased or decreased PTH levels in patients undergoing PD increase the occurrence of peritonitis risk, and the underlying possible pathophysiological mechanisms are still unclear. Thus far, to the best of our knowledge, there are only a few existing cohort studies that attempted to understand the associations between serum PTH and peritonitis in patients undergoing PD. A cohort study that included 270 patients who had PD revealed that, after adjusting for limited confounders, the multivariate analysis showed that lower serum PTH levels (with 150 pg/ml as a category) were independently associated with peritonitis in incident PD patients (55). Another retrospective, observational study showned that the unioncombinations of low PTH levels with either high Ca levels or with low/normal P levels wereas a considerable risk factors of for the occurrence of first episode of peritonitis in patients with undergoing PD (56).

In this study, we found that there was no statistical significance in the association between the lower PTH (tertile 1 or <150 pg/ml) group and peritonitis episodes after multivariate adjustment, although the lower iPTH tertile (tertile 1) was associated with 3-year peritonitis risk in the Kaplan–Meier survival analysis. The sensitivity analysis further showed the J-shaped association between serum iPTH and risk of first episode of peritonitis. Determining the target range for iPTH control in patients on PD remains difficult, and the underlying mechanisms by which elevated PTH leads to an increased risk of peritonitis are still poorly understood. However, there are some possible explanations for this phenomenon. Firstly, excess PTH may disturb the ability of the beta cells to improve insulin secretion appropriately, causing insulin resistance through a calcium-dependent mechanism (57, 58). It has also been demonstrated that the pancreatic β-cell function is relatively more impaired in patients with severe hyperparathyroidism on hemodialysis (59). Alterations, including glucose disorders, cause a state of metabolic syndrome in the body, which can cause susceptibility to peritonitis (60–63). Secondly, hyperparathyroidism aggravates hematopoietic dysfunction, inhibits erythropoiesis, and increases erythrocyte osmotic vulnerability, while muscular toxic effects lead to dysfunction and increased energy expenditure (64, 65), which worsen the fragile nutritional status of patients undergoing PD. Thirdly, increased PTH levels can lead to myelofibrosis and cardiac fibrosis in ESRD and may mediate cardiovascular fibrosis and apoptosis through the TGF-β signaling pathway, resulting in a hyperinflammatory state (66–68). Last but not least, as mentioned above, some of the different drugs used to treat SHPT, such as calcimimetics and vitamin D analogs, may have potential interactions between PTH and peritonitis. Moreover, PTH itself is considered a uremic toxin, and previous studies have confirmed that chronic exposure to higher PTH levels is associated with reduced T-lymphocyte proliferation, impaired immunoglobulin production, and immune dysfunction, while the individual immune system is strongly associated with the development of peritonitis in patients on PD (69–71). Further research is required for a better understanding of the mechanisms involved in this association.

There are some limitations in the present study. Firstly, this was a single-center, retrospective study. Ideally, a prospective study should be conducted for the purpose of averting the analysis bias associated with retrospective studies. Secondly, despite extensive adjustments for confounding factors, some factors that may affect the occurrence of peritonitis have not been taken into account, such as seasonality, training, and socioeconomic status, among others (27, 72, 73). Lastly, the clinical data, including the serum PTH levels, were baseline values for one measurement only. The lack of data from multiple measurements may lead to missing dynamic change factors. It should also be ensured that unnecessary bias is avoided.

In conclusion, higher serum iPTH levels are associated with an increased risk of peritonitis episodes in patients treated with CAPD. According to our findings, the deleterious effects associated with PTH appear to transcend its beneficial effects. Our results provide helpful data regarding the control of the serum PTH levels in patients on CAPD. The underlying mechanisms remain unclear, and further prospective studies with larger sample sizes are needed to confirm this relationship.

## Data availability statement

The original contributions presented in the study are included in the article/**Supplementary Material**. Further inquiries can be directed to the corresponding authors.

## Ethics statement

The studies involving human participants were reviewed and conformed to the ethical standards of the Research and Clinical Trial Ethics Committee of the First Affiliated Hospital of Zhengzhou University. Written informed consent for participation was not required for this study in accordance with the national legislation and the institutional requirements.

## Author contributions

ZZ and ZL designed the study, ZZ, DuL, GL, JC and SP collected and analyzed the data, ZZ and QY wrote the manuscript. WDL, JD, and ZL reviewed and revised the manuscript. All authors contributed to the article and approved the submitted version.
