# Corrigendum: Relationship between serum iPTH and peritonitis episodes in patients undergoing continuous ambulatory peritoneal dialysis

**DOI:** 10.3389/fendo.2023.1208563

**Published:** 2023-05-10

**Authors:** Zihao Zhao, Qianqian Yan, Duopin Li, Guangpu Li, Jingjing Cai, Shaokang Pan, Jiayu Duan, Dongwei Liu, Zhangsuo Liu

**Affiliations:** ^1^ Department of Integrated Traditional and Western Nephrology, The First Affiliated Hospital of Zhengzhou University, Zhengzhou, China; ^2^ Research Institute of Nephrology, Zhengzhou University, Zhengzhou, China; ^3^ Henan Province Research Center for Kidney Disease, Zhengzhou, China; ^4^ Key Laboratory of Precision Diagnosis and Treatment for Chronic Kidney Disease in Henan Province, Zhengzhou, China

**Keywords:** continuous ambulatory peritoneal dialysis (CAPD), parathyroid hormone, peritonitis, end-stage renal disease (ESRD), hazard ratio (HR)

In the published article, an author name was incorrectly written as Zhangzuo Liu. The correct spelling is Zhangsuo Liu.

The authors apologize for this error and state that this does not change the scientific conclusions of the article in any way. The original article has been updated.

