# Supplementary material for: Relationship between serum iPTH and peritonitis episodes in patients undergoing continuous ambulatory peritoneal dialysis
Source: Front Endocrinol (Lausanne). 2023 Mar 27;14:1081543. doi: 10.3389/fendo.2023.1081543 (PMC10083419; doi:10.3389/fendo.2023.1081543)

Supplement Table 1. All baseline demographic and clinical characteristics.

|  | **Total** | **Teritle 1** | **Teritle 2** | **Teritle 3** | **p value** |
| --- | --- | --- | --- | --- | --- |
|  | **(N=582)** | **(N=194)** | **(N=194)** | **(N=194)** |  |
| Sex, men | 349 (60.0%) | 109 (56.2%) | 123 (63.4%) | 117 (60.3%) | 0.347 |
| Age (years) | 45.1±11.5 | 46.4±11.4 | 46.7±11.1 | 42.1±11.4 | <0.01 |
| SBP (mmHg) | 140±18.2 | 137±18.6 | 140±15.4 | 143±19.9 | 0.011 |
| DBP (mmHg) | 87±12.4 | 85.5±12.2 | 86.8±11.0 | 88.7±13.7 | 0.042 |
| Smoker, yes | 47 (8.1%) | 19 (9.8%) | 14 (7.2%) | 14 (7.2%) | 0.561 |
| Drinker, yes | 32 (5.5%) | 9 (4.6%) | 13 (6.7%) | 10 (5.2%) | 0.651 |
| BMI (kg/m^2^) | 23.4±4.83 | 22.8±4.81 | 23.2±4.70 | 24.3±4.87 | <0.01 |
| **Comorbidities** |  |  |  |  |  |
| Hypertension, n(%) | 507 (87.1%) | 162 (83.5%) | 179 (92.3%) | 166 (85.6%) | 0.027 |
| Diabete, n (%) | 71 (12.2%) | 29 (14.9%) | 14 (7.2%) | 28 (14.4%) | 0.034 |
| CVD, n (%) | 97 (16.7%) | 38 (19.6%) | 27 (13.9%) | 32 (16.5%) | 0.324 |
| Hepatitis.B, n (%) | 35 (6.0%) | 11 (5.7%) | 10 (5.2%) | 14 (7.2%) | 0.674 |
| Edema, n (%) | 278 (47.8%) | 81 (41.8%) | 91 (46.9%) | 106 (54.6%) | 0.038 |
| **Medications use** |  |  |  |  |  |
| EPO (%) | 436 (74.9%) | 142 (73.2%) | 149 (76.8%) | 145 (74.7%) | 0.713 |
| Iron supplements (%) | 428 (73.5%) | 138 (71.1%) | 144 (74.2%) | 146 (75.3%) | 0.632 |
| Phosphorus lowering drugs (%) | 374 (64.3%) | 145 (74.7%) | 115 (59.3%) | 114 (58.8%) | <0.01 |
| PTH-regulating drugs (%) | 346 (59.5%) | 95 (49.0%) | 101 (52.1%) | 150 (77.3%) | <0.01 |
| Osteotriol | 260(44.7%) | 80(41.2%) | 89 (45.9%) | 91(46.9%) |  |
| Calcimimetics (Cinacalcet) | 4(0.7%) | - | - | 4(2.1%) |  |
| Other vitamin D analogues | 87(14.9%) | 15(7.7%) | 17(8.8%) | 55(28.4%) |  |
| UA-lowering drugs (%) | 102 (17.5%) | 42 (21.6%) | 31 (16.0%) | 29 (14.9%) | 0.174 |
| Statins (%) | 56 (9.6%) | 14 (7.2%) | 25 (12.9%) | 17 (8.8%) | 0.147 |
| **Dialysis-related data** |  |  |  |  |  |
| Laparoscopy (%) | 372 (63.9%) | 113 (58.2%) | 126 (64.9%) | 133 (68.6%) | 0.1 |
| Ultrafiltration (mL/day) | 400 [-1500, 2500] | 420 [-900, 2500] | 400 [-800, 2000] | 400 [-1500, 2300] | 0.86 |
| 24 h Urine output (L) | 200 [0, 2100] | 200 [0, 2100] | 200 [0, 2100] | 200 [0, 2100] | 0.71 |
| Total Weekly Kt/V | 2.09±0.31 | 2.11±0.32 | 2.07±0.29 | 2.09±0.31 | 0.469 |
| RRF(mL/min/1.73 m^2^) | 3.59±1.57 | 3.5±1.48 | 3.65±1.68 | 3.62±1.55 | 0.603 |
| **Laboratory variables** |  |  |  |  |  |
| WBC count (×10^9^/L) | 6.14±1.99 | 6.32±2.32 | 6.12±1.75 | 5.98±1.84 | 0.245 |
| Neutrophils (×10^9^/L) | 4.18±1.79 | 4.36±2.08 | 4.18±1.61 | 4.01±1.64 | 0.162 |
| lymphocyte (×10^9^/L) | 1.30±0.66 | 1.34±0.89 | 1.27±0.53 | 1.3±0.49 | 0.552 |
| monocyte (×10^9^/L) | 0.46±0.20 | 0.48±0.22 | 0.445±0.18 | 0.439±0.18 | 0.07 |
| Eosinophils (×10^9^/L) | 0.20±0.18 | 0.16±0.12 | 0.206±0.18 | 0.216±0.21 | 0.006 |
| Basophils (×10^9^/L) | 0.03±0.04 | 0.04±0.07 | 0.03±0.02 | 0.0335±0.02 | 0.567 |
| RBC count (×10^12^/L) | 3.33±0.80 | 3.52±0.82 | 3.25±0.7 | 3.37±0.78 | <0.01 |
| Hematocrit (L/L) | 0.31±0.07 | 0.32±0.07 | 0.304±0.07 | 0.3±0.08 | 0.01 |
| Hemoglobin (g/L) | 102±22.6 | 106±22.7 | 101±23.3 | 98.5±21.1 | <0.01 |
| Platelet (×10^9^/L) | 190±69.3 | 195±70.2 | 186±71.3 | 189±66.2 | 0.409 |
| FPG (mmol/L) | 4.94±2.2 | 4.91±2.07 | 4.93±2.46 | 4.97±2.04 | 0.957 |
| CD4+ count | 580 [44.0, 6490] | 568 [102, 6490] | 567 [44.0, 5410] | 596 [104, 2460] | 0.481 |
| CD8+ count | 428 [56.0, 6010] | 444 [100, 1530] | 425 [56.0, 5720] | 426 [88.0, 6010] | 0.861 |
| CD3+ count | 870 [57.0, 6020] | 964 [149, 3620] | 822 [130, 6020] | 811 [57.0, 2820] | 0.022 |
| Phosphorus (mmol/L) | 1.76±0.51 | 1.63±0.49 | 1.82±0.53 | 1.84±0.47 | <0.01 |
| Corrected calcium (mmol/L) | 2.15±0.26 | 2.25±0.28 | 2.1±0.25 | 2.09±0.23 | <0.01 |
| Potassium (mmol/L) | 4.31±0.74 | 4.30±0.74 | 4.36±0.79 | 4.28±0.70 | 0.532 |
| Natrium (mmol/L) | 141±4.16 | 140±4.25 | 140±4.27 | 141±3.89 | 0.018 |
| Serum Cl (mmol/L) | 98.7±5.23 | 98.7±4.99 | 99±5.78 | 98.4±4.89 | 0.629 |
| Serum Mg (mmol/L) | 1.02±0.18 | 1.03±0.18 | 1.03±0.17 | 1.01±0.18 | 0.331 |
| CO_2_ (mmol/L) | 25.0±4.02 | 25.3±4.12 | 24.5±4.02 | 25.2±3.89 | 0.133 |
| Serum Fe () | 14.5±6.68 | 14.2±7.04 | 14.3±5.9 | 14.9±7.06 | 0.598 |
| UIBC () | 27.1±15.70 | 27.4±23.70 | 26.8±9.53 | 27.1±9.54 | 0.919 |
| TIBC () | 40.6±8.14 | 39.5±8.21 | 40.4±8.01 | 41.9±8.06 | 0.012 |
| CRP (mg/L) | 13.4±31.4 | 14.5±32.8 | 13±31 | 12.7±30.4 | 0.824 |
| Esr (mm/h) | 42.3±30.8 | 40.1±29.9 | 41.8±30.7 | 44.9±31.9 | 0.298 |
| iPTH (pg/mL) | 262 [8.20, 2760] | 110 [8.20, 189] | 262 [189, 339] | 478 [340, 2760] | <0.01 |
| BUN (mmol/L) | 23.2±8.6 | 21.9±7.89 | 24.1±9.63 | 23.7±8.06 | 0.032 |
| Creatinine (mg/dL) | 10.65±4.32 | 836±289 | 979±478 | 999±329 | <0.01 |
| Uric acid (mmol/L) | 375±95.9 | 368±108 | 385±94.8 | 372±83.5 | 0.205 |
| TP (g/L) | 60.2±8.77 | 58.9±9.75 | 60.5±8.32 | 61.3±8.01 | 0.022 |
| Albumin (g/L) | 35.9±6.25 | 34.8±6.46 | 35.9±6.16 | 36.9±5.97 | 0.005 |
| ALT (U/L) | 20.3±19.6 | 21±22.6 | 18.7±15.3 | 21.3±20.0 | 0.355 |
| AST (U/L) | 19.3±12 | 20.1±14.7 | 18.2±8.72 | 19.5±11.8 | 0.268 |
| ALP (U/L) | 74.0 [8.00, 1510] | 65.0 [19.0, 1510] | 73.0 [24.0, 781] | 89.0 [8.00, 513] | <0.01 |
| Tbil (μmol/L) | 6.39±4.87 | 6.43±5.83 | 6.35±4.35 | 6.39±4.28 | 0.988 |
| Dbil (μmol/L) | 2.8±2.14 | 3.03±3.23 | 2.8±1.46 | 2.58±1.04 | 0.11 |
| Cholesterol (mmol/L) | 4.46±1.74 | 4.56±1.92 | 4.28±1.04 | 4.54±2.06 | 0.218 |
| Triglycerides (mmol/L) | 1.43±0.84 | 1.44±0.88 | 1.4±0.79 | 1.44±0.84 | 0.837 |
| HDL (mmol/L) | 1.13±0.34 | 1.12±0.32 | 1.11±0.35 | 1.15±0.36 | 0.524 |
| LDL (mmol/L) | 2.81±0.92 | 2.85±0.98 | 2.74±0.86 | 2.84±0.91 | 0.453 |
| **ECG and data** |  |  |  |  |  |
| RVDs (mm) | 16.5±1.92 | 16.4±1.74 | 16.5±1.97 | 16.5±2.03 | 0.873 |
| IVS (mm) | 10.9±4.01 | 10.7±1.71 | 11.2±6.54 | 10.9±1.63 | 0.555 |
| LVDs (mm) | 49.8±6.16 | 48.8±5.82 | 50±6.37 | 50.5±6.18 | 0.024 |
| LAD (mm) | 34.6±6.23 | 33.8±6.06 | 34.8±6.22 | 35.3±6.35 | 0.043 |
| EF (%) | 61.1±5.36 | 61.7±4.57 | 61.4±5.45 | 60.3±5.92 | 0.03 |
| QT Interval (ms) | 392±57.8 | 393±40.6 | 386±72.9 | 396±55.1 | 0.234 |
| QTc (ms) | 445±36.1 | 439±33.8 | 445±37.6 | 450±36.1 | 0.008 |
| HR (bpm) | 77.3±12.4 | 76.4±11.7 | 78.2±13.1 | 77.5±12.5 | 0.374 |
| PR Interval (ms) | 151±23.1 | 150±23.8 | 154±24.0 | 150±21.4 | 0.174 |
| ST.T changes, yes (%) | 210 (36.1%) | 72 (37.1%) | 63 (32.5%) | 75 (38.7%) | 0.418 |

Supplement Figure 1. The Kaplan-Meier survival curves with risk table.


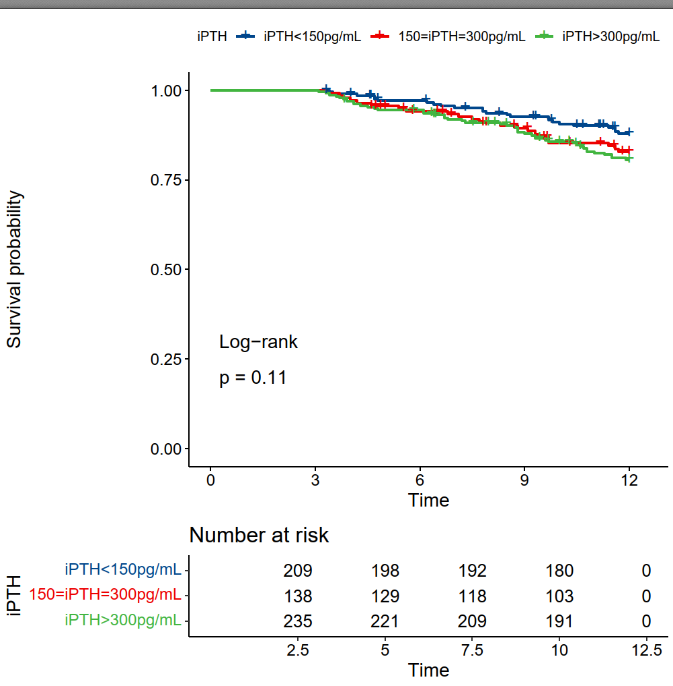
 At 1- year


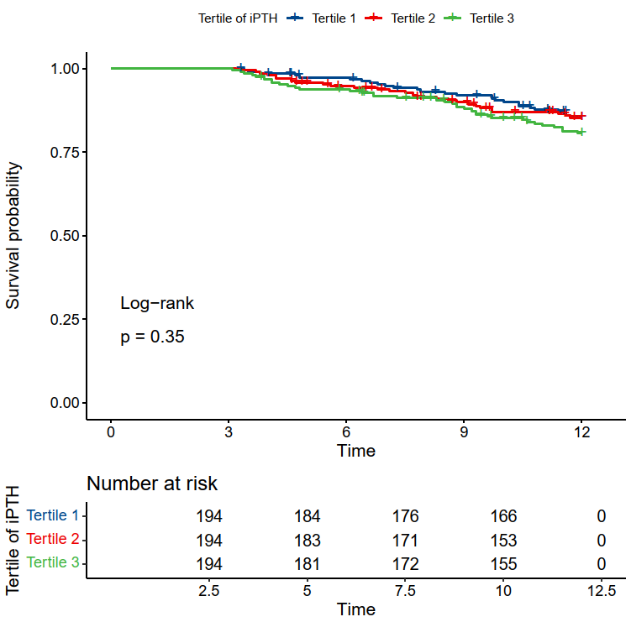


At 3- year


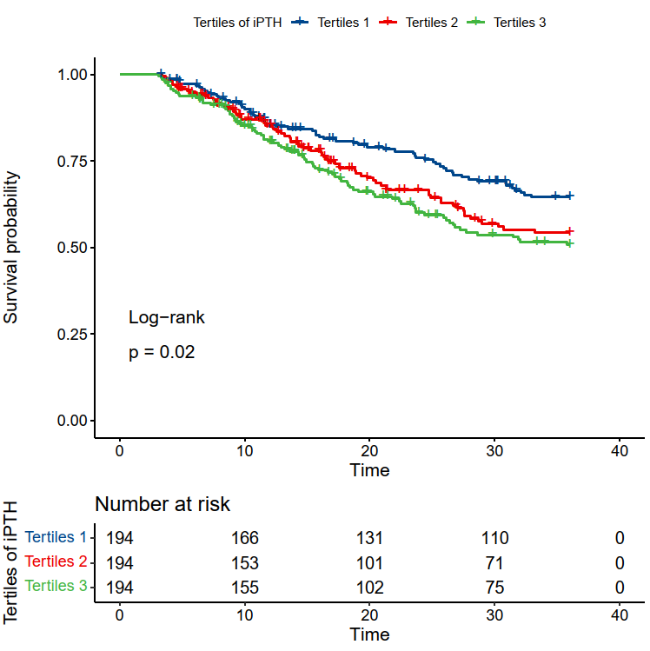

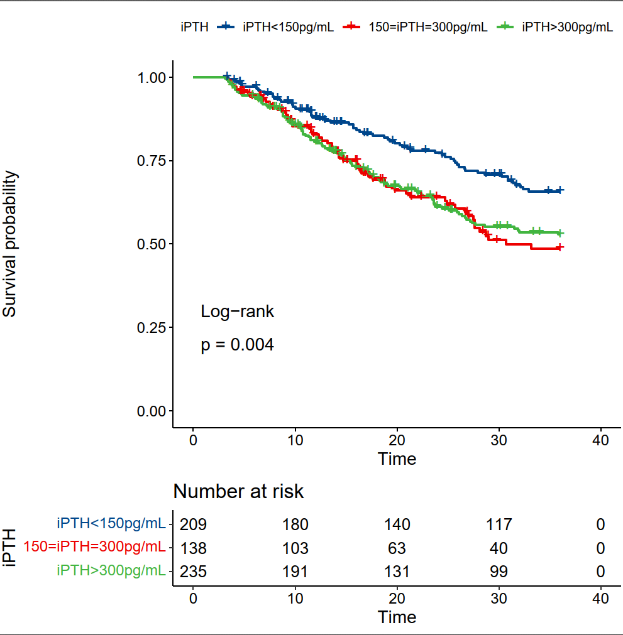


Supplement Figure 2. Pearson correlation analysis between every two baseline clinical data variables.


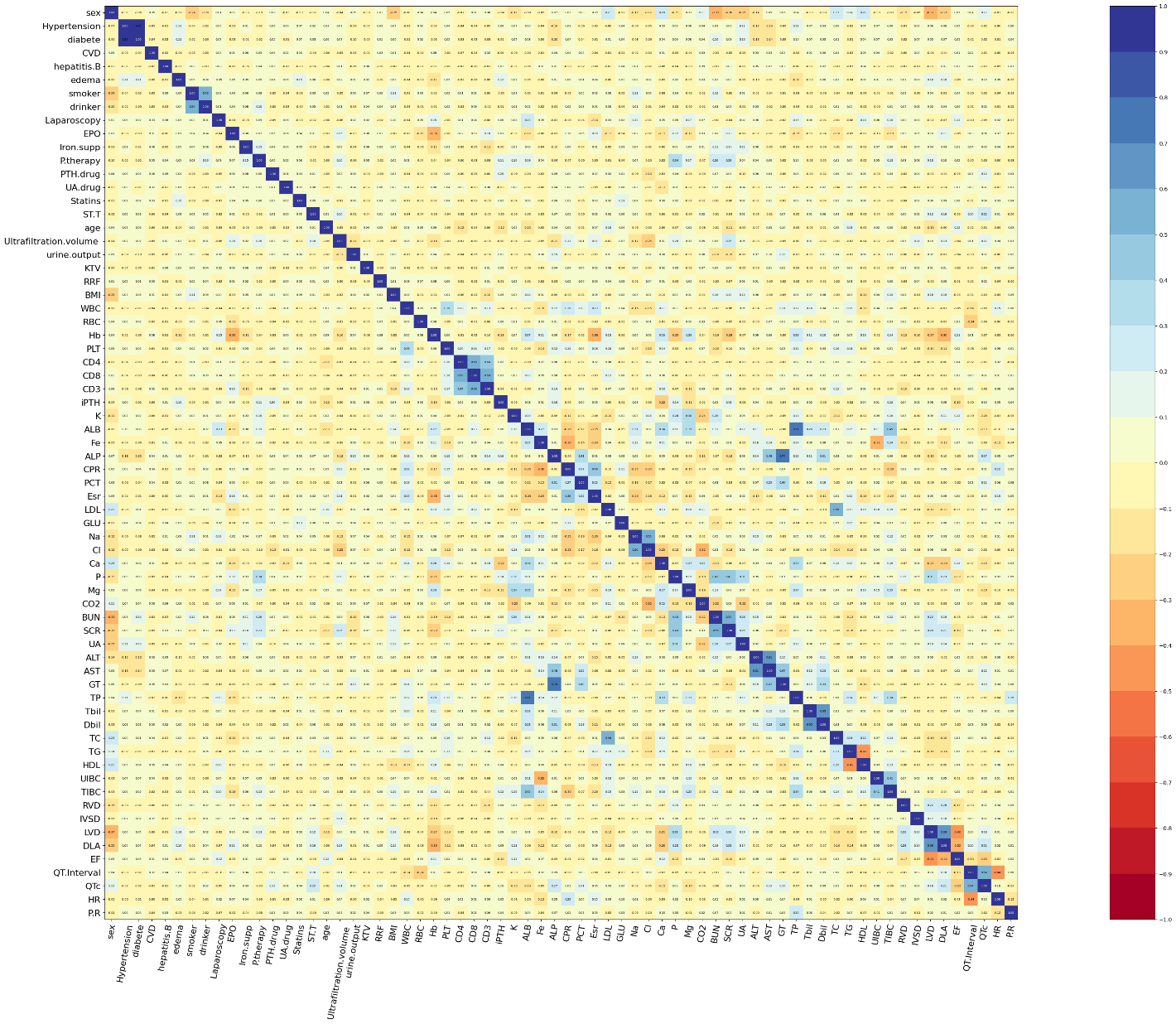


Supplement Figure 3. Martingale Residuals of fully-adjusted Cox model


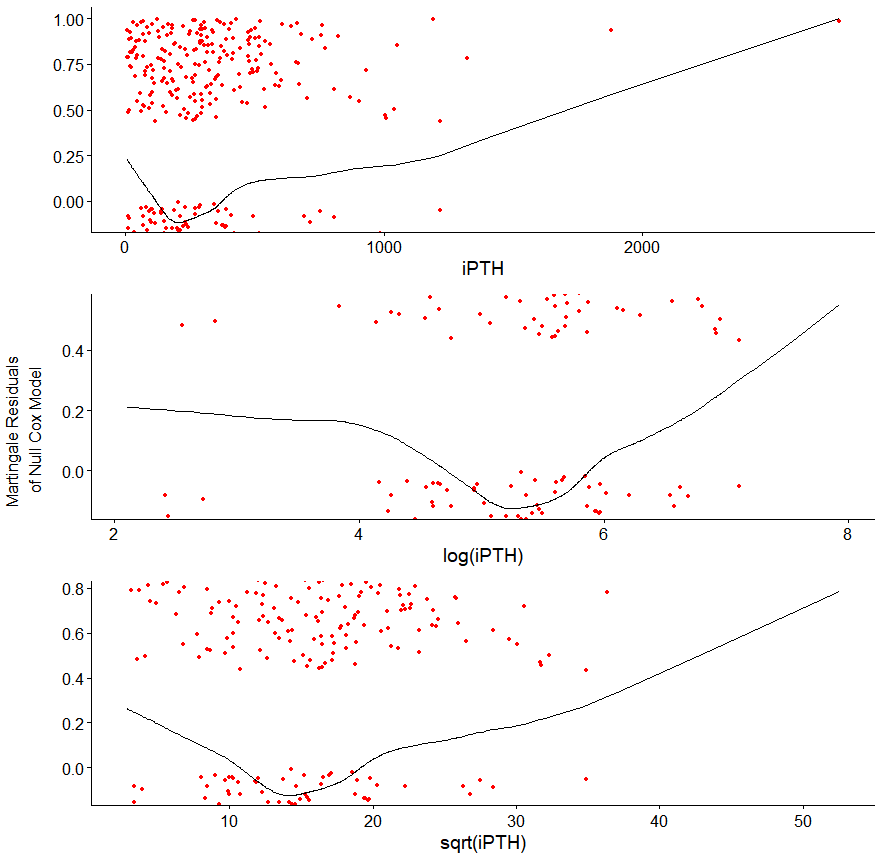

Supplement: Supplementary file 1 [file DataSheet_1.docx]
